# Supplementary material for: Measuring adverse events following hip arthroplasty surgery using administrative data without relying on ICD-codes
Source: PLoS One. 2020 Nov 5;15(11):e0242008. doi: 10.1371/journal.pone.0242008 (PMC7644076; doi:10.1371/journal.pone.0242008)
Supplement: S1 Appendix — (DOCX) [file pone.0242008.s001.docx]

## Appendix: Full technical description of the model development

## Training and selection of models

Before the training of the models, we randomly split the data set. One third of the patients was used as a holdout set that we only used in the final test of the best performing model (figure 1). The remaining two thirds of the patients were used as a training set for training and fine tuning of the models. During training we used 10-folds cross validation to measure and control for over-fitting.

We tested the following models: random forests, logistic regression with and without natural splines, support vector machines and neural networks with three different structures. The neural networks were fully connected networks that had the same type of input layer design using 18 input variables. We used three different network designs for the hidden layers:

- shallow: 1 hidden layer (6 neurons)
- deep: 5 hidden layers (9, 6, 6, 4 and 4 neurons),
- wide: 2 hidden layers (18 and 9 neurons).

We started the training with a dataset that included all variables. The importance of the different variables was evaluated by the generated importance in the output from the random forest model and with the use of the rms package with the lrm function for logistic regression models we tested the relative importance of the different variables with the Chi-Square test. We repeated the model training after removing the least important variables. LOS consisted of two variables, the LOS of the orthopaedic admission and LOS of the index admission.

We tried to tune the hyperparameters for some of the models (random forest and logistic regression), but in the most cases the default settings performed the best. We run the neural networks with 20 to 20 000 epochs and learning rates between 0.001 and 0.3.

## Validation of models

In the final test, we used the whole training set to train the model and the trained model was used for prediction for the holdout set. We made a prediction for each selection group. The sensitivity and specificity in the groups were multiplied by the group proportion (group size in population/total population) and summed, this yielded the adjusted sensitivity and specificity. The final model was evaluated against the holdout set. For the code-based model, the sensitivity, specificity and Youden’s index for the code-based model on the holdout data was calculated using the same method as on the training data.

## Performance metrics

We compared the models with the code-model by measuring sensitivity, specificity and Youden Index (sensitivity + specificity – 1).[1] For intermodal comparisons, we relied on the area under the receiver operator characteristic curve (AUC). The receiver operator characteristic (ROC) is created by plotting a curve of the different classification thresholds on the true positive and false positive rates. The AUC is the two-dimensional area under this curve. This curve could not be calculated for the code-model because the result from this model are dichotomous and does not contain any thresholds, we therefore used AUC during the model training and Youden Index for the validation of the final model.

## Software and Packages

We used R 3.5.1 for all statistics. We used the stats package for logistic regressions and the rms package (v.5.1-2) and the contrast function for calculating odds ratio and 95% confidence interval (CI) for age and LOS. The graphs were created using ggplot2 (v. 3.0.0). We used the ranger package (v.0.10.1) for all random forest models. We used the e1071 (v.1.7-0) package for support vector machines. We used the R interface for Keras (v.2.2.0) with TensorFlow (v.1.9) as backend for all neural network modelling. We scaled all continuous variables (0-1). To find the most accurate setup for the neural network we used the tfrun package which enabled us to test multiple different networks. All results were put in a data frame and we could choose the network with the highest accuracy to continue with. Result tables were created using the htmlTable package (v.1.12).

## References

1 Youden WJ. Index for rating diagnostic tests. *Cancer* 1950;**3**:32–5.
