# Supplementary material for: Measuring adverse events following hip arthroplasty surgery using administrative data without relying on ICD-codes
Source: PLoS One. 2020 Nov 5;15(11):e0242008. doi: 10.1371/journal.pone.0242008 (PMC7644076; doi:10.1371/journal.pone.0242008)
Supplement: Attachment — Submitted filename: Response to Reviewers.docx [file pone.0242008.s003.docx]

**Question:** 6. Review Comments to the Author

Please use the space provided to explain your answers to the questions above. You may also include additional comments for the author, including concerns about dual publication, research ethics, or publication ethics. (Please upload your review as an attachment if it exceeds 20,000 characters)

Reviewer #1: The manuscript is adjusted according to my comments. I consider this manuscript ready for publication.

Reviewer #2: I agree with the authors in relation to my Q12. No further change needed in the revised manuscript.

In relation to line 121 in revised manuscript. I would ask the authors to add the definition of "suffering" to the text as defined in A to my Q13, and likewise with "inevitable consequences" as defined in A to Q14.

**Answer:** Thank you for this constructive comment. We have added the suggested definitions.
